# 557. Effects of an Intervention to Improve Antibiotic Use in Dental Clinics Associated with an Academic Safety Net Institution

**DOI:** 10.1093/ofid/ofaf695.166

**Published:** 2026-01-11

**Authors:** Katherine C Shihadeh, M I chael Deaney, Margaret M Cooper, Kimberly A Meyers, Scott Hamilton, Timothy C Jenkins

**Affiliations:** Denver Health, Denver, CO; Denver Health, Denver, CO; Denver Health, Denver, CO; Denver Health Medical Center, Denver, Colorado; Denver Health, Denver, CO; Denver Health, Denver, CO

## Abstract

**Background:**

According to the CDC, dentists prescribe about 10% of all antibiotics in the U.S. These antibiotics may not be indicated or may be prescribed for longer durations than needed. This study aims to compare antibiotic use in a network of dental clinics before and after an intervention to optimize antibiotic use.

**Figure:**
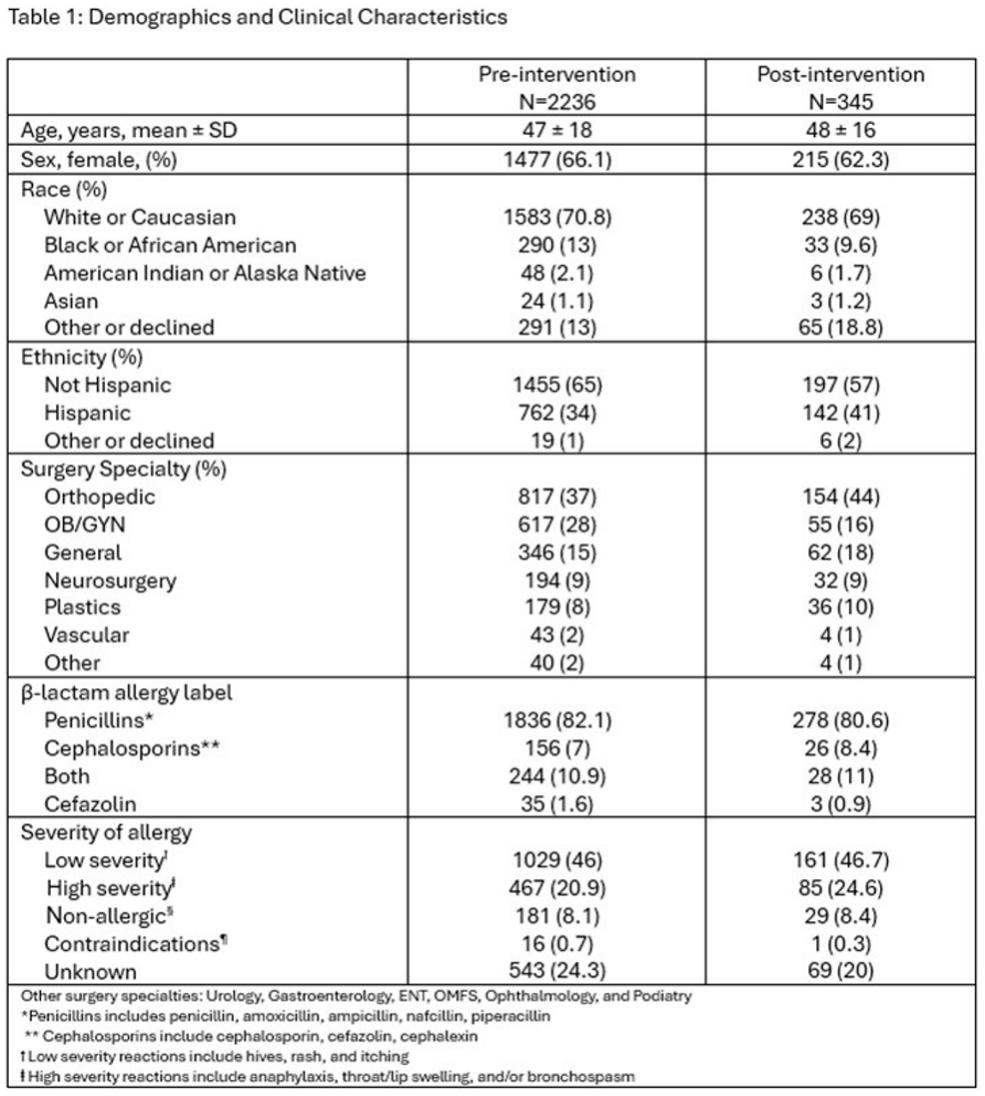
Demographics and Clinical Characteristics

**Figure:**
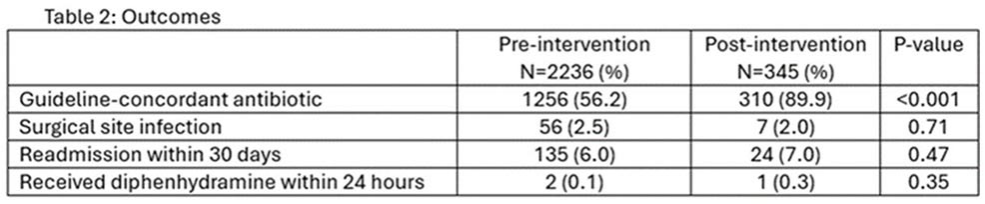
Outcomes

**Methods:**

This quasi-experimental study included adult patients who had a visit to one of 7 dental clinics in an academic, integrated healthcare system from 7/1/2022-11/30/2023 (pre-intervention) and 7/1/2024-3/31/2025 (post-intervention). A random sample of 200 visits that resulted in an antibiotic prescription was reviewed in each group. Prophylactic antibiotics were excluded. The intervention included engaging a dentist peer champion, presenting an hour-long educational session that was required by all dentists, and developing and disseminating an institutional guideline that was in accordance with the American Dental Association Guidelines that addressed when antibiotics are indicated, use of short (≤ 5-day) antibiotic courses, and avoidance of clindamycin in the setting of a penicillin allergy. The primary outcome was the proportion of dental visits where an antibiotic was prescribed. Secondary outcomes were the median duration of therapy, the proportion of antibiotic prescriptions for 5 days or less, and the proportion of patients prescribed clindamycin.

**Results:**

A total of 1,874 antibiotics were prescribed at 114,231 visits (1.6%) in the pre-intervention group and 764 antibiotics were prescribed at 71,678 visits (1.0%) in the post-intervention group (p< 0.01), representing a 35% relative reduction in antibiotic use. Although the median duration of therapy was 7 days in each group, a 5-day course was prescribed significantly more often in the post-intervention group (14.5% vs 37.5%, p-value < 0.01). Use of clindamycin in the setting of a penicillin allergy occurred with similar frequency in both periods.

**Conclusion:**

A multifaceted intervention reduced the rate of antibiotic prescribing in a network of dental clinics by 35% and shortened prescribed durations of therapy.

**Disclosures:**

All Authors: No reported disclosures

